# Does Distributive Inequality Cause Relational Inequality? Evidence from a Survey Experiment

**DOI:** 10.1007/s10677-025-10512-0

**Published:** 2025-12-26

**Authors:** Alice Baderin, Lucy Barnes, Lindsay Richards

**Affiliations:** 1 Department of Politics and International Relations, University of Reading, 293 Edith Morley Building, Whiteknights, RG6 6EL Reading, UK; 2 Department of Political Science, University College London, 29-31 Tavistock Square, WC1H 9QU London, UK; 3 Department of Sociology, University of Oxford, 42-43 Park End Street, OX1 1JD Oxford, UK

**Keywords:** Inequality, Relational egalitarianism, Distributive egalitarianism, Social status, Empirically-informed political theory

## Abstract

Contemporary egalitarian theory has been shaped by a debate between distributive and relational perspectives. Relational egalitarians argue that equality is primarily about the character of our social and political relationships, rather than the pattern of distribution of goods. But they often also claim that distributive and relational ideals are connected in practice, because material inequality impairs our ability to stand as social and political equals. Using a survey experiment, we assess the impact of material inequality on relational equality. We show that priming income inequality increases perceptions of unequal social status; but it does not affect perceived equal political standing, or the extent to which respondents affirm the ideal of relational equality. We argue that this empirical approach yields deeper payoffs for conceptual and normative questions about relational equality; and it contributes to wider methodological debates about the role of survey data in normative political theory.

## Introduction

What is the link between material inequality and inequality in our relationships? Specifically, does the unequal distribution of material resources within a society lead us to fail to regard and treat one another as equals? We present results from a survey experiment that investigates the causal connections between material and relational dimensions of inequality. Our research agenda stems from a prominent debate within egalitarian theory about the nature of equality: Should equality be understood in distributive terms? Or should egalitarian attention be focussed instead on the character of our social and political relationships? Whilst some relational egalitarians argue that the distributive paradigm mistakes the fundamental point of equality, they often also claim that the two ideals are connected in practice, because material inequality impairs our ability to stand as social and political equals. For example, at higher levels of distributive inequality, relative material disadvantage is increasingly likely to become a source of social stigma. On a strong version of this view, it is a ‘deep social fact’ (O’Neill 2008, 2010) that material inequality is corrosive of the egalitarian character of relationships.

We present a representative sample of UK adults with information highlighting material inequality and assess the impact on attitudinal measures of relational equality, as compared to a control group. Priming material inequality increases perceptions of unequal social standing, particularly for those with lower incomes. These results confirm the broad assumption, in the philosophical literature, that material inequality undermines relational equality. However, we also show that the connections are complex, in ways to which theorists have not been sensitive. Whilst our material inequality treatment has negative effects on relational equality measured in terms of self-perceived equal social status, we find no impact on perceptions of equal political standing, or on the extent to which respondents affirm the value of relational equality.

By subjecting political theorists’ empirical assumptions to more careful scrutiny, we generate payoffs for both normative theory and empirical social science. First, we respond to the call for a ‘serious psychology of egalitarianism’ (Scheffler 2005, p. 24), by providing a more nuanced picture of the empirical connections between distributive and relational equality. Second, we contribute to the emerging body of quantitative research on the social effects of material inequality. Approaching the empirical literature through the lens of relational equality gives us an organizing framework for a set of studies that have hitherto been conceptualized in disparate terms; and our experimental approach provides a valuable complement to existing cross-national evidence. Third, we show how investigating the material preconditions of relational equality offers opportunities to refine, and to test the nature of our commitment to, the relational ideal itself. Whilst there is no *direct* route from data about the conditions for realizing relational equality to claims about the value or meaning of the ideal, empirical evidence is an important (and underused) input into conceptual and normative debate. More generally, we argue that survey data can productively be used to assess the social psychological assumptions that commonly operate in the background of normative theory; we contrast this with a prominent alternative way of thinking about the role of public opinion in egalitarian political theory.

We begin by explaining the theoretical background to our study: clarifying our conception of relational equality and situating our work in relation to diverse philosophical accounts of the distributive-relational equality nexus. We then provide a brief overview of existing empirical evidence, before presenting our own experimental approach and results. Finally, the paper draws out the philosophical significance of our inquiry: for questions about how we should conceptualize and value relational equality; and for broader methodological debate about the role of public opinion data in political theory.

## Theoretical Framing

### What Is Relational Equality?

Relational egalitarians maintain that something went wrong in the debates about distributive equality that came to dominate egalitarian theory in the late twentieth century. In Elizabeth Anderson’s words, in focussing on the pattern of distribution of goods (of whatever sort), egalitarians lost sight of the ‘point of equality’, which is instead to ‘create a community in which people stand in relations of equality to others’ (Anderson 1999, pp. 288–289; O’Neill 2008; Scheffler 2003). Although united in their call to redirect egalitarian attention towards the character of our relationships, relational theorists differ on a range of issues concerning the value and meaning of ‘relating as equals’. For example, is relational equality instrumentally or non-instrumentally valuable? Is it a constraint to be honoured, rather than a good to be promoted?^1^ What does it mean, more specifically, to stand in relations of equality to one another? For the purposes of our empirical inquiry, we adopt a (in philosophical terms) relatively modest understanding of relational equality, as the absence of generalized status hierarchy.^2^ On this view, the basic relational egalitarian commitment is to a society in which no one is persistently looked down on or treated as inferior on the basis of some aspect of their social role or group membership: a community ‘not marked by status divisions such that one can place different people in hierarchically ranked categories’ (Miller 1997, p. 224). Such a commitment is violated, most starkly, in caste-based or racist societies. For example, in discussing the Brown v. Board of Education ruling, Anderson (1999b) highlights how racial segregation in education undermined relational equality, independently of its impact on black students’ wellbeing: ‘It would still have been wrong to brand them as inferiors, as the system of racial segregation did … Such action is wrong on account of the principles of contempt or inferiority that it expresses’. Relational inequality is also embodied in group-based practices of deference – think of labourers in the sixteenth century expected to bow or remove their hats when encountering members of the gentry – and, in the contemporary context, in systematic patterns of misrecognition based on age, gender, social class, or disability. For example, Bidadanure (2021, p. 105) highlights the infantilization of elderly people through ‘elderspeak’ as a less well recognized inegalitarian mode of relating: ‘Perhaps an older woman on the bus is being referred to by other passengers as “so cute” or “adorable” … Or perhaps a healthcare professional uses the pronoun “we” instead of “you” as in: “Do we want our lunch now?” or “How are we feeling today?” in a way that denies the patient a sense that they are seen as an agent’. On our view, relational equality demands, at a minimum, the elimination of the kinds of persistent status hierarchies manifested in these cases. However, it does not, as a conceptual matter, require the presence of close or solidaristic social connections (cf. Wolff 2015a, p. 24). Insofar as relational egalitarianism is intended as an account of the demands of *equality*, we find it helpful to distinguish the non-hierarchical quality of relationships from other potentially valuable characteristics of social bonds.^3^

The anti-status hierarchy principle is an appropriate place to start in an empirical investigation of the connections between distributive and relational equality, both because it represents a common core of more expansive accounts of relational equality, and because it is the notion that egalitarians often seem to have in mind when they claim that distributive inequality undermines relational equality in practice. However, relational egalitarianism is a multi-dimensional idea, and it is important to acknowledge further components that lie beyond the reach of our study. Most significantly, relational egalitarians are typically concerned not only with status, but also with power relations (e.g. Scheffler 2005, p. 17; Anderson 2008b, pp. 144-5; Schemmel 2021).^4^ They variously oppose situations of domination, in which some are in a position arbitrarily to interfere with the choices of others, and exploitative relations that enable some to take unfair advantage of others.^5^ We are sympathetic to the view that relational equality, in a fuller sense, encompasses opposition to certain relations of power or command, as well as to status hierarchy. However, assessing the mechanisms through which material inequality generates inegalitarian power dynamics is beyond the scope of this study; we can detect these processes only indirectly, insofar as they are reflected in survey respondents’ status attitudes.

Whilst we set aside the power dynamics that constitute an important element of relational equality, our empirical approach reflects the multidimensionality of the relational egalitarian ideal in two other senses. First, we take an expansive view of the ‘site’ of relational equality: we assume that the relational egalitarian ideal does not apply only or primarily to how states regard and treat their citizens, but also governs our interpersonal interactions (Fourie 2012, pp. 116–117; cf. Schemmel 2011). The narrower view risks losing much of the original impetus for the relational perspective, since it is the fundamental concern with how citizens regard and treat *one another* that distinguishes relational egalitarianism from many contemporary accounts of distributive justice (Garrau and Laborde 2015, pp. 49–50). Once we take this broader view of the site of relational equality, it is important to recognize that relational (in)equality may be revealed in different types of attitudes. Specifically, it seems that relational egalitarians should care about individuals’ other-regarding status attitudes (do I look down on others?); self-perceived status (do I think that others look down on me?); and certain normative beliefs (do I think that members of my community *ought* to relate to one another as equals?).^6^ Second, we respond to Lippert-Rasmussen’s observation (2018, p. 63) that to understand what it means to ‘relate as equals’, we must answer the question ‘equals along what dimension?’. Relational egalitarianism is plausible when it opposes somewhat persistent and generalized forms of hierarchy. Thus we need to unpack the general notion of relating as equals without setting ourselves against every instance of ranking, no matter how temporary or localized. Here we consider relational equality along two major dimensions: social and political. Social equality concerns our general standing as a member of society. Political equality centres on our status as members of a democratic community (see especially Anderson 1999): Do we stand as equals when it comes to determining the political decisions by which we live?

Thus we break down the concept of relational equality along two dimensions as shown in Table 1, which will provide an organizing framework for our empirical measures. The two grey cells have received less attention in the political theory literature that frames our study, and we do not examine them further here (though they have obvious referents in political science, for example in studies of commitment to democracy).

**Table 1:** Conceptual dimensions of relational equality

		Attitude type	
	Self-regarding	Other-regarding	Values
Social	Feeling of being included in society as an equal, being treated with respect in social interactions and not perceived as inferior.	Feeling that others are equal in social terms, treating them with respect in social interactions and not perceiving them as inferior.	Feeling that equal social relationships are desirable and rejecting social hierarchies
Political	Feeling of being included in political decision-making as a democratic equal.	Feeling that others are equal in political terms, respecting the input of others in political decision-making.	Feeling that equal and inclusive democratic politics is desirable, rejecting hierarchies of political treatment and influence.

Section 4 sets out the survey questions we use to operationalize each of these conceptual components of relational equality. Self-regarding social status is assessed using a battery of questions concerning whether individuals feel left out of society, feel the value of what they do is recognized, feel looked down on, and feel respected; as well as how they place themselves on a ‘status ladder’. Other-regarding status attitudes are gauged by a measure of respondents’ desire to distance themselves from other groups. Support for the value of relational equality is assessed through questions about whether it is a good or bad thing for certain groups to be at the top or to dominate society. Finally, we seek to measure respondents’ sense of their own political equality by asking whether they feel our system enables people like them to exert political influence.

As noted above, not everything relational egalitarians care about resides at the level of individual attitudes; relational equality typically also encompasses behavioural and expressive components, and we can imagine cases in which these elements come apart. For example, individuals may feel that they are looked down on – and may indeed be regarded as inferior – whilst nevertheless being treated as equals, or vice versa.^7^ Thus we seek to capture an important and widely shared part, not the whole, of the relational egalitarian ideal. All else equal, the greater the tendency for individuals to look down on members of other social groups, to think that others look down on them, and to fail to express support for the value of relational equality, the further we diverge from the ideal of a society of equals.

### Distributive and Relational Equality: Mapping Connections

Whilst distributive and relational egalitarians disagree about where the heart of egalitarianism lies, theorists on both sides of the debate have suggested a variety of ways in which the two commitments are connected. We can usefully distinguish four main accounts of these links within the philosophical literature:

1. Positive instrumental account: Material inequality causes relational inequality (or vice versa).
2. Negative instrumental account: Realizing (responsibility-sensitive) distributive equality will require us to take steps that undermine relational equality.
3. Constitutive account: Distributive (in)equality (in certain patterns or institutional forms) embodies or expresses relational (in)equality.
4. Procedural account: The meaning of distributive equality in any given context is to be determined through a process of decision-making among social equals.

According to the positive instrumental account, material inequality tends to undermine relational equality. Thus the prospects for realizing relational equality are likely to depend on setting some limits to income and wealth inequality. Different versions of this account recur across the literature on relational equality. For example, Scanlon (2018, p. 26) notes that ‘Economic inequality can … involve inequality of status if being poor means being unable to afford goods that are regarded as essential to being a respectable person’. Anderson (2008a, p .267) suggests that relational egalitarians have instrumental reasons to favour limits on top incomes, and to prefer that ‘individuals be crowded in the middle of the distribution’. And Scheffler (2020) claims that ‘extreme economic inequality undermines the ideal of a society of equals’. Most strongly, O’Neill (2010, p. 403) has argued that ‘philosophical accounts of the value of equality should proceed in light of appreciation of the ‘deep social fact’ that distributive inequalities tend to bring with them relationships of social domination, harms to individuals’ status [and] the breakdown of healthy fraternal social relations’.^8^ Importantly, the positive instrumental account centres on the connection between relational inequality and *material outcome inequality*: it points to a variety of ways in which gaps in economic resources can translate into status divides.

On a second account, efforts to realize distributive equality risk undermining relational equality. Most prominently, Wolff suggests that implementing responsibility-sensitive forms of distributive equality would require individuals to reveal information about their own lack of talent, in ways that would be experienced as shameful, and that would therefore be inimical to the kinds of respectful relationships required by relational equality (Wolff 1998). Wolff’s ‘shameful revelation’ account centres on the kinds of intrusive policy measures necessary to ascertain whether individuals are responsible for their own relative disadvantage. In an alternative version of the negative instrumental argument, Moles and Parr (2019) suggest that ‘score-keeping’ in personal relationships, in the way necessary to achieve strict distributive equality (responsibility-sensitive or not), would be corrosive of a sense of respect or equal standing among the parties to those relationships.

According to a third view, the connection between distributive and relational equality is not (only) causal, but constitutive. For example, Schemmel (2021, p. 232) argues that a Rawlsian version of relational egalitarianism, grounded in an ideal of society as a cooperative scheme among equals, implies ‘intrinsic reasons of an expressive kind in favour of limiting distributive inequality in socially produced goods. On one version of the constitutive view, material gaps beyond a certain size embody unequal regard (for critical discussion, see Cohen 2012, pp. 199–200). More strongly, we might hold that *any* departure from distributive equality, normatively understood, also constitutes a violation of relational equality, conceived as an ideal of mutual justification. A relationship in which I have more than my fair share is one in which I cannot properly justify my share to you, and I therefore fail to stand in justificatory community with you (Brighouse and Swift 2014, 27; Moles and Parr 2019, pp. 138-9). Fourth, and finally, there is a procedural picture of the distributive-relational inequality connection in some of Scheffler’s work, when he suggests that the proper meaning of distributive equality in a given context is to be determined through a process of decision-making among social equals (Scheffler 2015).

We have sketched out a complex web of potential connections between distributive and relational equality. These accounts are distinct but not mutually exclusive. Indeed, we could imagine them coming together in a particular case. Consider, for example, a disabled individual in UK who is subject to a ‘Work Capability Assessment’: an investigation of her level of impairment and ability to work that will determine her access to benefits. If the WCA process is stigmatizing, then we see the negative instrumental picture in action: the pursuit of responsibility-sensitive distributive equality undermines respectful relations.^9^ Now suppose that her relatively low income leaves her unable to afford goods or services necessary to take part in social life on terms of equality; she is also subject to ‘positive instrumental’ dynamics whereby material inequality undermines relational equality. Finally, proponents of the constitutive or procedural views might plausibly apply their arguments to this case. Perhaps the degree of material inequality generated by the welfare system expresses unequal regard towards welfare recipients, independently of its effect on status attitudes. And Scheffler could point to the ways in which welfare policy-making falls short of the ideal of a process of decision-making among social equals.

How does our study bear on these diverse pictures of the connections between distributive and relational equality? Our aim is to assess the positive instrumental view, specifically the version of that account on which the causal relationship runs from material to relational inequality.^10^ As noted above, the positive instrumental account concerns the connection between relational inequality and material outcome inequality. It might be objected then that we do not address the relational effects of *distributive equality* properly understood, which might include a commitment to responsibility-sensitivity, and/or a concern with the distribution of non-material goods. We are happy to concede this point: we are concerned with a claim about the relational consequences of material inequality that plays an important role in the philosophical literature, as well as raising empirical issues of independent significance for sociology and political science. However, our results may speak indirectly to the relational effects of distributive inequality normatively understood. Most theories of distributive equality imply that increased material inequality *relative to where we are now* would be detrimental in egalitarian terms. Thus, insofar as our experiment takes current levels of material inequality as a baseline, it bears on theories of distributive equality that do not take equality of material outcome to be the ultimate egalitarian goal.

Whilst the positive-instrumental account is widely shared among egalitarian philosophers, there are varying accounts of the nature and strength of this relationship. One important area of disagreement concerns which *patterns* of material inequality undermine equal relationships. Need relational egalitarians concern themselves only with distributive inequalities that leave those at the bottom below a material threshold, such that they cannot function as social equals? Notably, whilst Anderson sometimes suggests that a commitment to relational equality gives us reason to compress the gap between top and bottom incomes, elsewhere she draws sufficientarian distributive conclusions (for the sufficientarian account, see Anderson 1999, p. 321, 325). Relatedly, does inequality at the top end of the material distribution matter in status terms? Or can relational egalitarians afford to be sanguine about the 1% since, as Scanlon (2018, 37) suggests, ‘their life does not set any norms of expectation’ for the wider population? As well as testing the broad assumption that material inequality has negative relational egalitarian effects, we will address this debate within the positive instrumental camp, by presenting respondents with varying information highlighting different patterns of inequality.

Alternative philosophical accounts of the distributive-relational equality connection lie outside of the scope of this study.^11^ However, we note that those who argue that certain forms of material inequality embody or express relational inequality tend *also* to assert instrumental connections. For example, as well as pressing a version of the expressive view, Schemmel argues that there are instrumental relational egalitarian reasons to limit income and wealth inequalities. For mixed views such as Schemmel’s, empirical evidence might not immediately shift their picture of the distributive demands of relational equality. For example, there may be constitutive reasons to continue to oppose material inequalities that empirical research suggests are relationally inconsequential. Nevertheless, a more nuanced account of the instrumental requirements can help to clarify the grounds on which certain distributive inequalities are to be resisted. Relational egalitarians have sometimes failed sharply to distinguish constitutive and instrumental objections to distributive inequality; a tendency that can contribute to discussion of the distributive inequality-relational inequality connection becoming detached from empirical evidence. For example, in a recent philosophical paper, Heilinger (2024, p. 619) develops an account of the distributive conditions ‘that are not only necessary, but sufficient to provide a sound distributive basis for relational equality’. However, having emphasized that the ‘reason for which relational egalitarians should be concerned about distributions is their *instrumental* impact upon relations (2024, p. 621; emphasis added), he proceeds to move quickly between instrumental and constitutive objections to various material inequalities, without acknowledging the different kinds of evidence required to uphold these arguments. For example, he suggests that it ‘undermines, even violates’ relational equality when the wealthy enjoy superior healthcare, even if all have sufficient provision (Heilinger 2024, p. 624).^12^ Closer engagement with empirical research is useful insofar as it forces relational egalitarians to clarify the respective role of instrumental and constitutive/expressive concerns in justifying their resistance to material inequality.^13^

## Relational Egalitarian Effects of Material Inequality: Current Evidence

In support of the positive instrumental view, relational egalitarians have pointed to various mechanisms through which material inequality might threaten relational equality. Three accounts – the ‘consumption patterns’, ‘signalling’ and ‘system justification’ views – are particularly prominent in the philosophical literature. On the first narrative, economic inequality leads to divergent lifestyles which, in turn, mark out some as socially inferior (Scanlon 2018, p. 29). According to the ‘signalling’ account, the materially disadvantaged will interpret the unequal distribution of resources as a sign that their interests are not properly taken into account, and thus that they are not regarded as equals. Thus unequal distributions can undermine relational equality even if they lead neither to significant gaps in consumption, nor to hierarchical patterns of interaction (Moles and Parr 2019, p. 137). Finally, the ‘system justification’ view suggests that beliefs about group superiority or inferiority will be increasingly relied upon to rationalize material inequality at higher levels (Scheffler 2020).

What does current evidence say to these claims about the material inequality-relational inequality connection? Whilst the language of relational equality is confined to the philosophical literature, there is a wide range of empirical work, within sociology, political science and psychology, that addresses overlapping ideas. Here we briefly review research that speaks, in turn, to each of our four conceptual components of relational equality (self-regarding social status, other-regarding social status, support for relational equality, and self-regarding political status).

First, there is an important body of work on the social effects of income inequality that bears on relational equality as self-perceived social status. Sociologists have long been interested in the consequences of economic inequality for individuals’ perceptions of their own social standing. Sennett and Cobb’s classic *Hidden Injuries of Class* (1993) demonstrated that a working-class position was not simply an economic experience, but one shaped by powerlessness and feelings of inferiority. Social status and hierarchy remain central to the later epidemiological literature linking material inequality to health outcomes. Specifically, economic inequality is argued to intensify concerns about status and exacerbate social hierarchy, leading to increased stress and poorer health (Wilkinson 1996; Wilkinson and Pickett 2009, 2018). Importantly, Wilkinson and Pickett claim that material inequalities heighten status concerns even among the rich, who tend to make negative upward comparisons.^14^ Support for this ‘status anxiety hypothesis’ comes from evidence that in more economically unequal countries, agreement with the statement ‘people look down on me because of my job situation or income’ is higher (Layte and Whelan 2014; Delhey and Dragolov 2014; Layte 2012), and subjective social status is lower (Lindemann and Saar 2014; Schneider 2019).^15^ Self-perceived social status has also been identified as the mechanism linking material inequality to populist voting. Gidron and Hall (2020) report that where economic inequality is higher, perceptions of being social valued – for some groups in particular – are undermined. Engler and Weisstanner (2021) argue that higher economic inequality creates a more pronounced social hierarchy, driving support for radical right parties who commit to defending traditional status boundaries.

These studies, across sociology and political science, provide evidence of correlations between income inequality and indicators of social standing that resonate closely with the ‘self-regarding social status’ component of relational equality. However, the robustness of these relationships is still contested. For example, drawing on European data, Steckermeier and Delhey (2019) report that the country-level association between income inequality and feelings of inferiority disappears once GDP is controlled. Further, whilst Layte and Whelan (2014) find that feelings of status inferiority are higher among individuals at all income ranks in more unequal countries, income inequality does not *intensify* the relationship between income position and status.

There is less research that speaks directly to the second (other-regarding social status) and third (support for the ideal of relational equality) elements of relational equality. In terms of other-regarding attitudes, a large literature in sociology connects income inequality to levels of social mixing and social trust (E.g., Lancee and van der Werfhorst 2012; Steijn and Lancee 2011). However, this research does not allow us to differentiate general social disengagement from lack of cohesion that results specifically from regarding others as inferior; and it is the latter that is directly relevant to relational inequality. Similarly, there is a relative lack of evidence when it comes to the impact of material inequality on public support for the ideal of relational equality (our third component), notwithstanding an extensive body of work in political economy that investigates the effects of income inequality on *distributional* ideals (e.g. Rueda and Stegmueller 2019). Psychological research on ‘social dominance orientation’ examines the affirmation of values that overlap substantially with relational equality (e.g. Pratto et al. 1994). However, variation is typically regarded as a matter of personality, and as a cause that explains other outcomes, rather than an outcome potentially shaped by the prevailing material context.

Our final dimension of relational equality concerns individuals’ perceptions of their own political standing. Here a large political science literature considers the consequences of material distributions for democratic inclusion. At the level of the polity as a whole, greater equality may help consolidate and maintain democratic institutions (e.g. Houle 2009). For individuals, higher inequality is associated with lower overall voter turnout, and a wider gap between low and high-income groups (Schäfer and Schwander 2019); and with lower and more unequal levels of broader democratic engagement, including political interest and discussion, and civic engagement (Lancee & van der Werfhorst 2012; Solt 2008).

We have briefly reviewed some diverse empirical literatures that are usefully related to one another through the lens of relational equality, and together offer some support for the expectation among philosophers that material inequality leads to relational inequality. However, in the best-developed area (self-regarding social status), the findings are mixed. This may stem from the difficulty of effectively identifying the impact of material inequality in cross-national observational data, when many other features of the national context also differ. But equally, there are several factors that may mute or complicate the transmission of material to relational inequality. First, public understanding of the extent and shape of material inequality is limited (Gimpleson and Treisman 2018). To the extent that the link depends on individuals’ awareness of economic disparities, these misperceptions render the relational consequences of *objective* material inequality less clear. There is also a tendency for individuals to make social comparisons with local reference groups (Irwin 2015; Condon and Wichowsky 2020), making it less obvious how *society wide* material inequality produces social hierarchy. Finally, relative material position exists alongside alternative potential bases of status differentiation such as age, gender, race and occupation. We would expect the material inequality-relational inequality link to be sensitive to the extent to which, in any given context, money is either *pre-eminent* or *dominant* in status terms (Miller 1995): Does material advantage outweigh other factors when we assign status? To what extent can money be converted into goods that confer status in non-monetary spheres? Particular cases suggest complexity and contingency in these connections. For example, relatively low levels of income inequality in Japan have coexisted with marked status hierarchy (Goldthorpe 2010).^16^

## Empirical Design

The difficulty of disentangling the impact of material inequality as a feature of observed contexts motivates our alternative experimental approach. Specifically, we use a between-subjects information provision survey experiment, with novel measures of different dimensions of relational equality as our outcomes. Respondents are randomly assigned to receive one of four types of information with equal probability. Three treatment groups see a figure and text describing different patterns of inequality. The control group is shown information about economic growth in an analogous format.^17^ The survey was fielded by YouGov to a nationally representative sample of over 3700 adults in the United Kingdom in November and December 2019.^18^ We focus on the UK since, along with the United States, it has seen large increases in income and wealth inequality over the past generation, and is relatively unequal. If material inequality translates into relational inequality, it is particularly important to identify these effects in high-inequality contexts.

Information provision experiments allow for the generation of exogenous variation in perceptions of real-world environments – such as the level of material inequality – when these phenomena cannot be directly changed (Haaland et al. 2023). This approach has been widely used to examine the attitudinal impact of economic inequality (for example, Cruces et al. 2013; Kuziemko et al. 2015). Most similar to our approach are experiments by McCall et al. (2017) and Trump (2017), who randomise the provision of information about increasing levels of inequality (McCall) or unequal pay between occupations (Trump), to investigate effects on attitudes towards economic inequality. McCall et al. find that inequality information increases the importance attributed to structural factors in determining economic opportunity. Trump identifies a just world effect, in which greater levels of inequality are deemed legitimate when respondents receive information about current high inequality.

We follow these studies in manipulating perceived levels of inequality through the provision of information; and we extend their reach by measuring the impact on relational rather than distributive attitudes.^19^ The price we pay for the greater causal precision offered by this experimental approach is a one-step remove from the real-life dynamics linking material and relational inequality. We are able credibly to identify the impact of perceptions of greater inequality on respondents’ relational attitudes; and many of the theoretical mechanisms for the transmission of material to relational inequality do run through individuals’ awareness of material inequality. But we cannot investigate effects of material inequality that are operating via other channels. For example, high levels of material inequality may lead to increasingly divergent lifestyles and reduced interaction between socioeconomic groups. This lack of contact might in turn drive prejudicial attitudes towards outgroups, a pathway that does not depend on individuals’ awareness of the extent of material inequality. Thus our approach seeks to isolate one important piece of the complex empirical puzzle around the relational effects of material inequality.^20^

### Determinants: Perceived Economic Inequality, Experimentally Manipulated

**Fig. 1:**
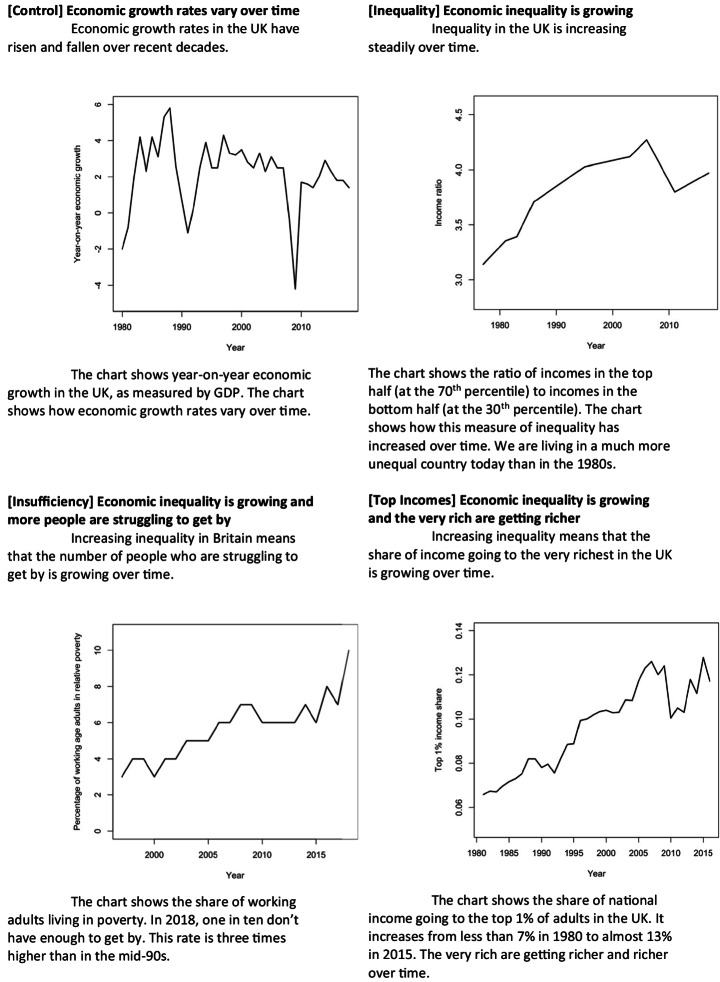
Information presented to control and treatment groups

Figure 1 shows the inequality treatments and the information given to our control group (which is designed to replicate the experience of being exposed to economic issues and to quantitative data). The three variations of the treatment highlight different patterns of inequality: inequality in general; inequality with insufficiency at the bottom of the distribution; and inequality driven by the increasing incomes of the very rich. Each group also sees a refresher text halfway through the survey, reminding them of the information in the figures.

The texts and figures are designed to be short and relatively neutrally framed, while providing the information in a way that maximises understanding among respondents (Haaland et al. 2023). However, attributing the effects of these compound and multidimensional presentations to the latent treatment of perceived material inequality requires two additional assumptions (Fong and Grimmer 2023). First, we must assume that other features differentiating the treatment texts and images do not drive our results. Buttressing this assumption is the high degree of similarity across treatments. All show trends over time, and both quantitative and qualitative presentations of economic data. The second assumption is that there are no interactions between the latent treatment and the method of delivering it. The three different presentations of inequality mitigate this concern. We include a manipulation check at the end of the survey to ascertain whether we have successfully generated differences in perceptions of material inequality between treatment and control groups. The treatments lead respondents to report systematically higher levels of inequality.^21^

### Outcomes: Relational Equality

As discussed above, the idea of relational equality can be decomposed into distinct attitude types across social and political domains. Table 2 summarizes the survey items that we rely on to create our four types of relational equality outcome. Most of these questions are well validated, and some are widely used in the sociological research on status inequality. We also include a new question to capture self-regarding social equality directly (‘When I’m dealing with other people, they respect me as an equal’). However, measuring relational equality as it resides in other-directed attitudes presents a challenge. Social desirability dynamics make eliciting direct expressions of ‘looking down on others’ extremely difficult in aggregate terms. Variation in measurement error (the degree to which people find such expressions acceptable) may also be large relative to variation in truly-held attitudes. Thus, we tap the other-regarding aspect of relational equality more indirectly, by probing participants’ desire for social distance from a variety of groups.^22^

**Table 2:** Empirical operationalisation of four dimensions of relational equality

		Attitude type		
		Self-regarding	Other-regarding	Values
Dimension	Social	**Own equal social status** I feel left out of society; I do not feel that the value of what I do is recognized by others; Some people look down on me because of my job situation or income; When I’m dealing with other people, they respect me as an equal; Self-placement on status ladder.	**Desired social distance from other groups** Would not want/would be happy to have neighbours like this: people a lot poorer than me people a lot richer than me unemployed people	**Commitment to the value of relational equality** Some groups of people simply inferior to other groups; Good thing that certain groups at the top and other groups at bottom; No one group should dominate in society.
	Political	**Own political inclusion** Political system allows people like me to have an influence on politics		

The construction of the four dependent variables from these survey items was specified in advance of the data collection.^23^ For each of the three social variables, we combine the multiple items into additive indices, subject to checks for reliability.^24^ This results in a scale of perceived own social status which runs from five to 25, and a scale of commitment to relational equality from three to 15. The other-regarding measures do not reach acceptable levels of reliability when combined, so (as per our pre-analysis plan) we revert to the use of the single item for desired distance to ‘people a lot poorer than me’. This scale runs from zero to ten.

The central hypothesis suggested by the philosophical literature (and receiving mixed empirical support) is that higher levels of material inequality drive higher levels of relational inequality. This should be the case for relational equality in both social and political dimensions, and as it resides in different attitude types. Thus, our primary hypotheses are that respondents in the inequality treatment groups will show:

- H1. lower levels of own equal social status.
- H2. lower levels of own political inclusion.
- H3. greater desired social distance from other economic groups.
- H4. lower levels of commitment to relational equality.

## Results: The Impact of Distributive Inequality on Four Relational Outcomes

**Fig. 2:**
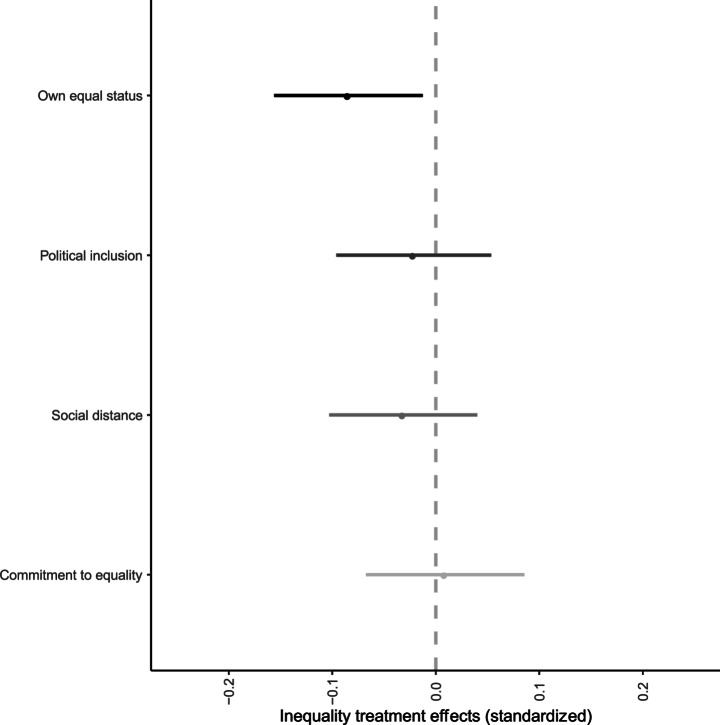
Standardised treatment effects of (combined) inequality treatment on different types of relational outcomes from models with controls (for age, gender, region and income). Horizontal bars are 95% confidence intervals

We find small but real and consistent effects on respondents’ perceptions that they are regarded and treated as a social equal. However, we find no evidence that material inequality affects perceived political standing, desire for social distance from the poor, or the tendency to endorse relational egalitarian values. Figure 2 shows the effects of the inequality treatments (combined) on each of these four main outcomes. The dependent variables are standardized, so each estimate reflects the effect of the treatment in units of a standard deviation.

The dark point at the top of the figure illustrates the treatment effect on own social status: a drop of just under one tenth of a standard deviation of this variable. In the original units, the difference is 0.35 points. For context, the gap observed between low- and high-income groups is 2.86 so the treatment effect is equal to 12% of the total observed difference between poorer and richer groups. For the other three relational equality outcomes, the estimated effects of receiving the material inequality treatments are smaller, and not statistically differentiable from zero. We note that this is not simply a case of imprecision, but that we identify relatively precisely estimated effects of zero for these outcomes.^25^

We also recover very similar effects across the inequality treatments, which highlight different patterns of inequality. There are three (non-exclusive) ways to interpret this result. First, it provides reassurance that our findings are not tied to specific details of the inequality presented. Second, it challenges the assumption, among some philosophers, that only material inequality involving insufficiency is consequential for relational egalitarianism. By showing that sense of equal status is damaged in a similar way by information highlighting inequality in general or runaway fortunes at the top, we add weight to the argument that relational egalitarians cannot be distributive sufficientarians. Third, it may be that there are real differences, but they are too small to be identified given the sample size, which was calibrated to identify differences between the control group and the combined treatment groups.

### Varying Effects Across Individuals

**Fig. 3:**
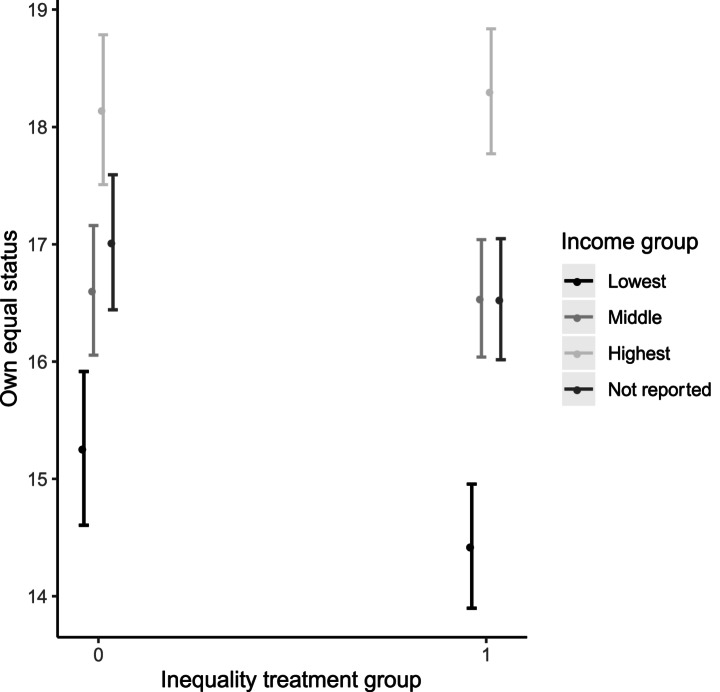
Predicted values of own equal status by income group and treatment status. Estimates from models including controls for age, gender, region. Vertical bars are 95% confidence intervals

Many of the theoretical arguments for the translation of material to relational inequality point to an effect driven by status harms to the less well off. This implies that the impact of our treatment on the perception of one’s own equal status should be more pronounced for those with lower incomes. Figure 3 shows the predicted outcomes for perceptions of equal social status for four income groupings: low, middle, high, and those who do not report their incomes.^26^ For the lowest income group, the decline in the equal status index under the inequality condition is 0.84 points, while the middle- and upper- income groups register little difference (a 0.07 point decline for the middle, a 0.16 increase for the rich). It is those with the lowest incomes who drive the negative treatment effects, as well as (to a lesser extent) those who do not report their income in the survey.

This result is consistent with the intuition that material inequality would be costly in status terms for those who are at the losing end of that inequality. But the absence of any negative impact among the better off stands in opposition to the narrative, most strongly associated with Wilkinson and Pickett (2009; 2018), that material inequality is bad for everyone’s sense of status. It also contrasts with the pattern reported by Layte and Whelan (2014) in the observational data, of across-the-board increases in status inferiority as inequality increases. We find, instead, a pivoting of the gradient between income and perceived equal social standing, such that higher inequality increases its slope.^27^

### Desire for Social Distance from the Rich

Our findings so far suggest that the damage to relational equality that is done by material inequality operates through an effect on individuals’ sense of their own social status; and this is primarily driven by those who are materially disadvantaged. There is an asymmetry in our results here, as we do not recover a parallel increase in respondents’ desire for social distance from the poor (the hypothesis we set out to test in our pre-analysis plan). Instead, we see a small but significant increase in the desire for distance from the rich (see Table A16 in the statistical appendix). In other words, whilst the materially less advantaged are increasingly likely to feel that others look down on them under conditions of inequality, heightening a sense of material inequality in fact seems to produce more inegalitarian relational attitudes towards the well-off. Specifically, seeing information about the extent of income inequality makes people less willing to have rich people as neighbours, by one third of a point on the original ten-point scale (one-tenth of a standard deviation of the outcome).

There is a question about the proper interpretation of this finding, given that we rely on a measure of desired social distance to gauge the extent to which respondents look down on other social groups.^28^ On the one hand, the observed effect might be driven by fears of gentrification, or by a perception that the rich are socially different; and these attitudes do not appear to be a direct affront to relational equality, understood as an anti-status *hierarchy* principle. We might also be picking up the impact on own social status via a more indirect route: perhaps material inequality leads respondents to want distance from the rich because they are more likely to believe that the rich look down on *them*. Equally, inequality may lead people to want to minimise social comparison with the rich to protect their own esteem (Condon and Wichowsky 2020) and our treatment may be eliciting these attitudes. On the other hand, previous research has highlighted negative perceptions of the rich under conditions of high inequality, including the sense that the wealthy are morally unworthy (e.g. Zitelmann 2020; Sherman 2017). Further empirical work is needed to better understand the dynamics underlying this aspect of our findings.

## Discussion and Philosophical Implications

What should we take from these results overall, as a response to the question: Does distributive inequality cause relational inequality? Our interpretation is that the answer is yes, but in quite narrowly defined terms that reinforce the importance of carefully specifying our empirical measures of relational inequality. Our results for the perception of one’s own social status are important, particularly for those with low incomes. An overall effect size that is over a tenth of the size of the difference between rich and poor overall, and more than twice that for the lowest income group,^29^ is meaningful evidence of a negative impact of distributive inequality on how people perceive their own social standing. This difference arises from a brief and unemotional presentation of information about the true extent of distributive inequality. To the extent that direct personal experiences, or repeated exposure to information about inequality further undermine equal social status, our results provide a lower bound to the effects in the real world. Moreover, and contra the assumptions of some relational egalitarians, we find no evidence that the negative status implications of material inequality are restricted to contexts in which inequality is bound up with insufficiency. We recover similar effects when highlighting inequality in general, or inequality driven by runaway fortunes at the top of the distribution. As discussed above, our contribution complements the observational studies that incorporate the holistic differences between high and low material inequality contexts, but at the cost of being unable to disentangle specific features of those contexts that drive relational outcomes.

A similar interpretation should be given to the null results we recover. We do not find effects of our information treatment on perceptions of political inclusion, desired social distance from the poor, or the endorsement of relational egalitarian ideals. This also holds within the different income groups.^30^ This may be because distributive inequality is less important for these kinds of relational outcomes, or because the effects run through pathways that are not captured by our experimental approach. This study represents a first effort to operationalize the complex philosophical ideal of relational equality in a multidimensional way. Our results open avenues for future research exploring the connections between material inequality and different types of relational outcomes.

What further philosophical implications follow from our empirical findings?^31^ We do not expect to prompt egalitarian philosophers to rethink their most basic commitments, especially since our overall results support a widely shared claim. However, we think that this type of empirical endeavour – in which we identify and test social psychological assumptions working in the background of normative theory – often has more subtle philosophical payoffs. In the next section, we identify two ways in which our study casts light on questions about the nature and value of relational equality. We then go on to situate our interdisciplinary approach in the context of a wider set of methodological conversations about the role of public opinion in political theory.

### The Meaning and Value of Relational Equality

#### Conceptual Clarification: New Questions for Relational Egalitarians

We have suggested that relational equality may be a useful organizing concept for empirical scholars of inequality. In mapping the terrain of status harms (or anxieties), political inclusion, social distance, and commitment to egalitarian values through their shared quality as dimensions of relational inequality, we provide a conceptual framework to synthesise some disparate literatures. This mapping encourages empirical researchers to be more precise about their concepts of interest, and offers them a language to articulate the normative underpinnings of their work. But there are less obvious conceptual payoffs in the other direction too, since operationalizing complex theoretical ideas can force to the surface conceptual issues that would otherwise remain hidden. In this way, the process of measurement is a valuable, and underused, tool for the task of conceptual clarification that is basic to philosophical endeavour.^32^ Two examples illustrate where our effort to capture relational equality through survey items proved conceptually revealing. First, it highlighted a neglected question about the different attitude types in which the ideal may reside in practice. Do self-perceived status, other-regarding attitudes, and popular value commitments each have a place, as our approach assumes? Is one component conceptually more core? Whilst theoretical work on relational equality has emphasized the multidimensionality of the relational egalitarian ideal, this type of attitudinal complexity has not been well recognized. These questions are made more pressing by our finding that the three attitude types do not respond equally to material inequality. How then should relational egalitarians think about a situation in which increased material inequality makes some people feel worse about their own status position, but does not, on the whole, lead to more exclusionary other-regarding attitudes, or reduce the tendency to affirm the ideal of relational equality?

Operationalizing relational equality also required us to assess how this philosophical concept intersects with existing empirical measures of related ideas. For example, we employed the widely used ‘status ladder’ question, which asks individuals to assign themselves a social ranking from 1 to 10. This measure maps closely onto the relational egalitarian concern about social hierarchy. However, it is less obvious what the relational ideal implies for the interpretation of different response patterns. In the maximally socially equal society, presumably everyone should place themselves at 5. But how should we compare, for example, a situation in which responses are distributed across 3, 5 and 7, with a context in which everyone says 7, or one in which everyone says 3?^33^ More generally, reflection on this survey item points to two neglected conceptual questions about relational equality: First, is relational equality about the absence of status *gaps*, or the tendency for people to place themselves in the *middle* of society? Second, is a general tendency for people to see themselves as superior in status terms less or more problematic than a shared sense of status inferiority? These questions have not, as far as we are aware, been addressed by relational egalitarians.

These examples illustrate what Ryan and Spinner-Halev (2022, 1121) have referred to as a ‘concretizing’ role for quantitative methods in political philosophy, in which leveraging survey data to assess philosophers’ empirical assumptions can generate indirect philosophical payoffs. Our examples show how the process of measurement can be conceptually revealing, independently of the results, because the discipline of translating abstract ideas into intelligible survey questions confronts us with new conceptual puzzles. Ryan and Spinner-Halev offer a further illustration, in which new survey data about status dynamics yields some pressing conceptual questions for relational egalitarians. Like us, they use an experimental approach to test an empirical assumption that is operating in the background of relational egalitarian theory. Specifically, they investigate whether the social status of a speaker directly affects the credence that listeners accord to her political views (as, they suggest, relational egalitarians tend to assume) or whether other factors are at work. Contra the relational egalitarian assumption, they find no evidence of a direct relationship between status and credence. However, respondents *are* more likely to dismiss the views of speakers with a poor communication style or a mode of communication that might signal low status. This result, in turn, exposes some deeper philosophical questions about the nature of relational equality and its connection with equal credence: ‘We might give more credence to those who communicate well— but does this pattern violate equality? … Would social egalitarians object to people holding talented orators in high esteem? How about talented chemists or philosophers?’ (Ryan and Spinner-Halev 2022, 1129).

#### Testing Normative Commitments: Putting Pressure on Relational Exclusivism

We have suggested that the application of survey methods may yield conceptual insights. Can an empirical approach also speak to normative questions about the place of relational equality within the egalitarian ideal? Our argument here starts from the broader observation that normative commitments sometimes gain force from background assumptions about the conditions under which they would be realized. Engaging in empirical work that potentially disrupts those assumptions can, therefore, offer valuable opportunities to rethink the nature of our commitment to the ideals themselves. Interrogating the distributive preconditions of relational equality, in the way we have sought to do here, is especially important for ‘relational exclusivism’ (Miklosi 2018): the view that distributions have no fundamental egalitarian significance independent of their relational implications (for example, Scheffler 2015, p. 2; Anderson 2008a, p. 242). On this view, the proper distributive commitments of egalitarianism are highly contingent on the relational consequences of alternative distributive arrangements.^34^ Our research agenda was motivated, in part, by the concern that it is too easy to be a relational egalitarian in this strong sense if we tell an empirical story whereby we also get a significant measure of distributive equality for free. Whilst some distributive egalitarians have objected that ‘relational egalitarianism vacates a large part of the terrain of distributive justice’ (Schemmel 2011, p. 369), our question, instead, was whether some relational egalitarians have attempted to hold onto this terrain via inadequately supported empirical claims. In particular, we worried that something like O’Neill’s ‘deep social fact’ account might work in the background of the theoretical literature, to insulate relational egalitarians from confronting potential gaps, or even trade-offs, between realizing these different dimensions of equality.

By holding this picture up to empirical scrutiny, we can question the assumption that distributive and relational equality are tightly connected in practice, and thereby put pressure on the claim that the normative significance of distributive equality is derivative of its relational consequences. For example, consider the possibility that we could effectively advance relational equality by equalizing distributions within reference groups, whilst remaining indifferent to large distributive inequalities between certain groups (cf. Rawls 1999, p. 470 on non-comparing groups). Or, more strongly, consider the potential to further relational equality by *increasing* material inequality and thereby disrupting the potential for status-threatening social comparisons (Condon and Wichowsky 2020; Paskov 2017). Would relational egalitarians be prepared to say that these distributive strategies represent unequivocal steps towards a more equal society? If not, what does this reveal about how they value relational and distributive equality?

As the discussion above illustrates, we may scrutinize certain normative commitments by reassessing their appeal in light of hypothetical stories about the conditions for their realization. But real facts can be a more powerful tool in normative inquiry: ‘sometimes … responses to actual facts reveal our principles better than our responses to hypothesized facts do, because the actual facts present themselves more vividly to us, and, too, they concentrate the mind better, since they call for actual and not merely hypothetical decisions’ (Cohen 2003, p. 227). Whilst our findings overall add weight to the positive instrumental account, two aspects of our data illustrate this deeper role for empirical investigation in normative thinking about relational equality. First, our finding that priming material inequality results in more exclusionary attitudes towards the rich runs counter to the dominant picture in the philosophical literature, in which the primary concern is for the negative impact of material inequality on the status of those at the bottom of the distribution. Probing reactions to this finding is therefore potentially revealing, as a tool for reflecting on the respective place of relational and distributive commitments within the egalitarian ideal. How troubling do we find it, in egalitarian terms, when material inequality activates a desire for social distance from the rich? Our suspicion here is that the relational egalitarian would be *more* concerned if these exclusionary attitudes were directed towards those who are also relatively materially disadvantaged; and this suggests that a concern for material inequality *is* doing some independent normative work.

More broadly, our results are illustrative of the complexity of the instrumental links between distributive and relational inequality. We find that these connections may vary according to the attitudinal component of relational equality, the domain, or the income group. The question then for relational exclusivists is whether they have the internal resources to accommodate judgements about how problematic, in egalitarian terms, these more nuanced patterns appear. If some of these associations intuitively seem worse than others for equality (e.g. because they leave those who are materially worse off at the bottom of a status divide), can we properly account for this in relational terms? Or do we need to fall back on claims about the non-instrumental significance of distributions? This challenge can be hidden by vague discussion of ‘less’ or ‘more’ relational equality under different distributive conditions. We suggest then that relational egalitarians should be more cautious about thinking simply in terms of ‘amounts’ of relational equality, which will vary according to the distributive context. Instead, they ought to engage with the kind of multidimensionality we begin to reveal in our study. Of course, the empirical evidence is not decisive in normative terms. But, in our view, properly engaging with the complexity of the empirical connections between distributive and relational equality adds weight to the case for a pluralist account of equality, according to which both distributive and relational commitments play a fundamental role.

Second, whilst we affirm the shared expectation that material inequality undermines status equality, we also intervene in a disagreement about which patterns of material inequality are consequential for relational equality. For relational egalitarians who have assumed – either explicitly or implicitly – that only inequality associated with insufficiency is problematic in status terms, our results are a prompt to recheck the credibility of their ideal under more demanding distributive preconditions. Does the appeal of the relational ideal remain unchanged when we substitute the expectation that, in practice, it will require attending to material gaps among those who have ‘enough’? As noted earlier, our claim about the normative significance of our survey data is a modest one. We cannot directly read off implications for the fundamental debate about the point of equality. However, evidence that unsettles our background empirical expectations can function as an epistemic tool for philosophers: encouraging us to think about how we value relational equality against the backdrop of revised assumptions about the circumstances for its practical realization.

In this way, the results of philosophically-driven empirical inquiry can function like (particularly forceful) thought experiments, helping to test and refine certain intuitions.^35^ But they can also be part of an iterative process of reasoning, in which empirical findings stimulate downstream philosophical thought experiments that could in turn give shape to novel empirical research. In our study, we address income-group difference in the *target* of negative other-regarding attitudes, finding that priming material inequality increases the desire to disassociate from the rich not the poor. But this result led us to reflect on the egalitarian salience of potential variation in who *expresses* such attitudes; something we did not initially set out to investigate. Compare, for example, a hypothetical situation in which negative attitudes towards the rich under conditions of material inequality are concentrated among the poor; and a context in which the rich too express greater desire for distance from the wealthy under conditions of material inequality. Which, if either, pattern would be preferable in relational egalitarian terms?^36^ In general, inter-group variation in other-regarding status judgements appears positive from the relational egalitarian perspective, since it suggests that status norms are less consensual. As Nath notes, an individual’s equal standing ‘can be compromised when some aspect of who she is, or what she does, invites negative appraisal on the basis of *shared* norms that prevail in a given social context’ (Nath 2020, p. 3; emphasis added). Yet, at the same time, associations between relative material advantage and other-regarding status attitudes appear intuitively troubling to us, insofar as they suggest that material gaps are cutting deeper into the formation of our relationships. In this way, our findings prompted a further line of reflection on inter-group differences in status attitudes that could, in turn, help to inform future empirical research.

### Methodological Implications: the Role of Public Opinion in Normative Political theory

We have outlined two ways in which our study, whilst primarily empirical in character, offers indirect insights into philosophical questions about the meaning of relational equality and its place within the egalitarian ideal. By presenting an extended example of integrating survey data into political theory, we also address broader methodological debates about the role of empirical evidence in normative inquiry. These conversations have ranged from abstract discussion of the relationship between facts and principles (Cohen 2003) to detailed accounts of the normative payoffs of specific forms of empirical data (for example, Zacka et al. 2021).

Our aim is to exemplify a productive model for integrating large scale survey data into political theory. The first step is to identify an empirical conjecture about popular attitudes that is working in the background of normative theory, and to translate this into a testable empirical hypothesis. Our focal claim – about the relational harms of economic inequality – is illustrative of a type of social psychological assumption that is widespread in contemporary political theory. Political philosophers commonly rely on claims about how social, political or economic circumstances will shape normatively salient self-perceptions or other-regarding attitudes. For example, theorists of multiculturalism maintain that particular forms of cultural belonging are an essential precondition of individual self-respect; some defences of free speech suggest that expressing our views helps us to crystallize our identity; and liberal nationalists assert that shared national identity fosters social and political trust. Such claims are often amenable to testing using survey methods that reveal systematic patterns in public attitudes.^37^ Indeed, this approach could fruitfully be applied to address philosophical claims about the distributive-relational inequality link that are outside of the scope of the present study. Consider, for example, the suggestion that, in order to uphold relational equality, it is necessary to impose tighter limits on inequality in certain types of goods, such as healthcare, relative to others, such as income (Heilinger 2024, p. 626, 629). Claims about the varying relational significance of distributive inequalities in different goods could similarly be investigated using survey experimental methods. The second step then is to bring relevant survey data to bear: generating new data where necessary, or reanalysing existing data where reasonable proxies for our philosophical concepts are available.^38^ Finally, there is reflection on the deeper payoffs: Have we brought any neglected conceptual questions to the surface in the course of our empirical inquiry? Do our results challenge philosophers’ expectations about what would be required in practice to realize an ideal, or what the consequences would be? If so, what role were those assumptions playing in the architecture of the normative theory? Does revising them prompt any rethinking about how we value the ideal itself?

It is useful to draw out the underlying shape of our approach, since it contrasts with a prominent alternative way of thinking about the contribution of public opinion data to egalitarian political theory.^39^ Specifically, it is commonly assumed, by both defenders and critics of more opinion-sensitive approaches to theorizing about social justice and equality, that the relevant data concerns popular normative attitudes. On this view, we use survey methods to study the public ‘as political philosophers’, investigating popular responses to the normative questions that divide political theorists. Elsewhere, we have called this the ‘congruence model’ of opinion-sensitive political theory (Baderin forthcoming), since the goal is to assess the extent to which a candidate normative theory matches the content of public opinion.^40^ This approach has been applied most prominently by David Miller in his defence of a pluralistic theory of social justice, which, in line with popular views, accords a significant place to considerations of desert. On Miller’s view (1999, 51), ‘a normative theory of justice … is to be tested, in part, by its correspondence with our evidence concerning everyday beliefs about justice’.

There has been extensive debate about the appeal of the congruence model, in particular whether it can make good on the promise to reconcile a more opinion-sensitive approach with the critical role of political theory in relation to prevailing attitudes and practices. In affording public opinion a larger role in political theory, do we inevitably lapse into a problematic kind of conservatism? (see Baderin et al. 2018). Our preferred approach side-steps this problem, since it does not involve ‘testing’ normative theory in a direct way, by comparing its content with wider public views. Instead, we leverage survey data to scrutinize philosophers’ background empirical conjectures about public attitudes. In doing so, we also address the developing field of experimental political philosophy. Political philosophy has been under-represented within the broader discipline of experimental philosophy. However, the most prominent strand of research has focussed on uncovering what principles of justice people will accept. By shifting attention away from popular justice beliefs and towards egalitarian theorists’ background social psychology, we seek to model an alternative approach to experimental political philosophy. This has important further implications for debates about why experimental research should form part of political philosophy. In particular, it suggests that the normative salience of survey data need not rest on any controversial commitment to ideals of publicity or stability (cf. Lindauer 2023, p. 3–7). Minimally, we need only agree that empirical assumptions are widespread in political philosophy, and that it is better to increase the accuracy of these claims. More ambitiously, we have sought to show that interrogating philosophers’ empirical hypotheses can also contribute to conceptual and normative debate.

This picture of the role of public opinion data can also usefully be situated in relation to a recent call for greater ‘robustness’ in political theory: for theories that are ‘capable of withstanding reasonable changes to their assumptions and to the cases in which their arguments apply’ (Kirshner and Spinner-Halev 2024, 1658). Just as empirical scholars assess whether their results are sensitive to reasonable alternative choices about their data – for example, the exclusion of outlying observations or the specification of different functional links between variables – political theorists should investigate whether their normative theories remain credible given reasonable changes to the cases or circumstances in which they apply. Consider, for example, G.A Cohen’s defence of socialism, which proceeds by drawing out the appeal of socialist principles in the context of a camping trip (Cohen 2009). In response, Cohen’s critics have pointed out how the credibility of his socialist ideals is bound up with specific features of the camping context – such as the unity of purpose among participants – that do not apply to wider social systems. Thus Cohen’s theory appears problematically ad hoc: ‘built by capturing how a subject does or should work under a limited set of conditions, the theory’s success may be contingent on those conditions, failing to remain credible when tested against the broader range of conditions and cases the author is targeting’ (Kirshner and Spinner-Halev 2024, 1660).

Perhaps we do not need detailed empirical evidence to trace normatively salient differences between the camping trip and other social contexts. However, empirical research will often play an important role in the pursuit of robustness in political theory, since what constitutes a *reasonable* alternative assumption will typically implicate complex empirical issues. Thus, our evaluation of the positive instrumental account can be viewed as a contribution to the wider task of increasing the robustness of relational egalitarian theory. First, our experimental data suggests that there is a reasonable alternative to the assumption, among some relational egalitarians, that relational equality is threatened only when inequality also involves insufficiency. And our suggestion to recheck the appeal of the relational egalitarian ideal under more demanding distributive preconditions echoes Kirshner and Spinner-Halev’s wider point: that we should assess the credibility of normative theory against reasonable rival assumptions about the conditions under which it is intended to apply.

Second, Kirshner and Spinner-Halev suggest that a philosopher might pre-empt a demand to demonstrate that her theory is robust to alternative assumptions by showing that ‘plausible alternative assumptions are actually less likely to characterize the situations she targets than the assumptions she uses’ (2024, 1663). From this perspective, evidence confirming the widely shared positive instrumental account is also useful. By generating novel experimental data to support this background claim, we also provide resources for relational egalitarians to respond to potential challenges to the robustness of their theory.

## Conclusion

Contemporary egalitarian political theory has been structured by a distinction between distributive and relational ideals. We have sought to approach the theoretical debate from a new angle, by investigating the background empirical assumption that material inequality causes relational inequality. Drawing on a survey experiment with a representative sample of UK adults, we find that material inequality does indeed undermine relational equality, a result that holds across different patterns of material inequality. These effects arise primarily via the negative effects of material inequality on the self-perceived social (but not political) standing of those in the lowest income groups. In contrast to the idea that ‘inequality is bad for everyone’ in status terms, we find no evidence that highlighting material inequality depresses sense of social status among the rich.

In investigating the causal links between material and relational inequality, we engage with a set of disciplinary conversations about inequality and social status that span political science, sociology and political theory. This endeavour yields payoffs for both empirical social science and normative theory. The concept of relational equality has provided us with an organizing perspective on a fragmented set of empirical literatures that have sometimes lacked conceptual precision. On the other hand, we have sought to show that moving beyond armchair social science can yield insights into conceptual and normative issues that are core to political theory. Our broader aim has been to exemplify a productive model of empirical political theory, on which we expose, and interrogate, the social-psychological assumptions that commonly lie in the background of normative theorizing.

## Data Availability

Replication files for this article are available through the Open Science Foundation: https://osf.io/bpvwr/files.

## Footnotes

1 On the value of relational equality, see Tomlin (2015). Our overall approach does not rest on any specific position in debates about why relational equality matters.

2 Here we follow Wolff (2015b) in defining relational equality in negative terms, although we will employ a combination of positive and negative indicators for the purposes of measurement.

3 This conceptual move has implications for the relevance of sociological work on the relationship between income inequality and social capital, social cohesion and social trust. This data is only indirectly relevant to our research question, since, whilst lack of social cohesion may be a cause of social hierarchy, it is not constitutive of relational inequality.

4 Anderson (2008b) distinguishes three main types of social hierarchy: standing, esteem and command. Our conception of relational equality maps most closely onto Anderson’s inequality of esteem.

5 Above we used Bidadanure’s example of infantilization to illustrate relational equality as status hierarchy, involving the expression of demeaning attitudes based on age. Insofar as infantilization also involves the denial of decision-making power to elderly people, this component again lies beyond the scope of our inquiry.

6 We do not treat individuals’ own sense of self-worth (as opposed to how they think that others regard them) as a conceptual component of relational equality. Whilst individuals may internalize status inferiority, on our view low self-respect is not constitutive of unequal *relationships*.

7 On equal regard versus equal treatment, see Cohen (2012, p. 197) and Voigt (2018, pp. 438 − 41). Relational equality plausibly also implicates implicit attitudes – Lippert Rasmussen (2018, p. 86). Capturing these attitudes is again outside the scope of our study.

8 O’Neill relies heavily on the work of Wilkinson and Pickett, suggesting that they have given us ‘an overwhelmingly strong empirical case for accepting the claim that this ‘deep social fact’ is, indeed, a fact’(2010, p. 403). But Wilkinson and Pickett themselves do not offer direct evidence of a material inequality-status inequality link, instead treating income inequality as a proxy for status inequality. The next section discusses some empirical work that has addressed this relationship more directly.

9 On experiences of the WCA as disrespectful, see, for example, Dwyer et al. (2020).

10 We do not rule out a causal effect in the other direction, for example because inegalitarian relational attitudes decrease support for redistribution (Thal 2020), but we do not directly assess this.

11 Different methods are needed to assess the negative instrumental argument; and constitutive claims are not open to direct empirical assessment. There is a further question about how the expressive meaning of institutional arrangements is to be determined, and whether empirical evidence can play a heuristic role. For discussion, see Voigt (2018, pp. 444 − 53).

12 It is difficult, then, to see where the weight of the argument lies when Heilinger (2024, p. 629) concludes that even relatively small inequalities in healthcare will ‘advance... a two-class society’. See also his discussion of limitarianism, which similarly moves quickly between the claim that extreme wealth *expresses* a form of disrespect incompatible with the relational egalitarian ideal, and more instrumental concerns (Heilinger 2024, pp. 623 − 24).

13 For relational egalitarians who deny that distributive patterns have independent egalitarian significance, we should be suspicious of any tendency immediately to fall back on constitutive arguments in the face of evidence challenging instrumental claims. See Sect. 6 for further discussion of the way in which empirical research puts pressure on relational exclusivism.

14 In contrast, Condon and Wichowsky (2020) emphasise that important psychological pressures – particularly under conditions of high inequality – push people away from making upward social comparisons, and segregated daily experience and media coverage of the rich make such comparisons practically difficult.

15 There appears to be a conceptual mismatch between status *anxiety* and relational *inequality*. However, status anxiety is most commonly operationalized via perceptions of inferiority, rather than feelings of worry about status. Thus much of this literature *is* directly relevant to our agenda. More generally, in the absence of any consistent conceptual repertoire in the sociological literature, we must look to details of the measurement strategy within individual studies to assess their relevance to relational equality.

16 For an account emphasizing this contingency, see Schemmel (2021; Chap. 8).

17 Randomisation yields roughly 945 respondents in each group. Our sample size implies that we are adequately powered to detect effects greater than 0.13 standard deviations in the outcome at conventional levels. Prior work with inequality information treatments yields effects of around 0.25 (on the judgment that inequality is a very serious problem (Kuziemko et al. 2015)) to 0.4 (on preferred levels of inequality (Trump 2018)). Full details of our design, measurement and pre-analysis plan, as well as the full statistical analyses for all results reported here, are available in an online appendix: https://osf.io/bpvwr/files.

18 All of the figures are based on real data. Ethical review was conducted by the University Reading, with approval granted on 9th October 2019. We were in the field November 22, 27–29, December 2, and December 13–16. Note that there was a general election in the UK on December 12, 2019. We avoided fielding surveys in the immediate run-up to the election, but our early respondents were participating in our survey during the campaign, and the later set of respondents in the immediate post-election context. Our treatment groups are balanced across and within waves and in general there are few differences between the samples of respondents before and after the election.

19 We have identified only one existing information provision experiment (Melita et al. 2021) that assesses the impact of material inequality on relational inequality with a non-representative sample. Further, where Melita and colleagues ask respondents how they *would* feel about their status in a hypothetical high inequality society (‘Bimboola’), we assess respondents’ actual status attitudes after exposure to real income inequality data.

20 A more general question about information provision designs is whether any effects arise from learning or priming (Haaland et al. 2023). For our purposes this does not matter: as long as the treated individuals are more aware of inequality, this provides the variation we need.

21 The effect sizes across all three types of inequality information are similar.

22 Note that we measure *desire* for distance, not actual social distance. So, whilst this measure takes us one step away from the concept of social hierarchy, it still avoids collapsing the distinction, emphasized earlier, between relational equality and social connectedness.

23 Details are provided in the statistical appendix.

24 We reverse item scales where necessary so that higher values are assigned in all cases to the expression of more egalitarian responses.

25 Note that while the estimates for political inclusion and for social distance are not statistically distinguishable from zero, and that for own equal treatment is, the magnitude of the political inclusion and social distance estimates are not significantly different from that for own equal status at conventional levels. Using the test statistic z = (b_1_-b_2_)/√(s_b1_^2^ + s_b2_^2^), p-values from the one-tailed test (that the negative effect on own social status is bigger) are 0.12 and 0.15 for political inclusion and social distance respectively.

26 The groups comprise 22 (lowest), 28 (middle), 17 (highest) and 34 (not reporting) % of the (weighted) sample respectively.

27 This pattern is consistent with Bartram’s (2022) longitudinal analysis of European data. However, Bartram finds that periods of higher inequality *boost* sense of status among the better off (contra Wilkinson and Pickett) whilst having little impact on lower earners.

28 On avoiding interaction with members of other social groups as an indicator of relational equality, see Voigt (2017).

29 The treatment effect of 0.84 for this group equates to almost three tenths of the low- to high-income own equal status difference.

30 See the statistical appendix for full results.

31 We thank an anonymous reviewer for encouraging us to draw out the philosophical and methodological implications of our study.

32 Perez (2020) offers a helpful typology of the usages of data in political theory. Our account of conceptual clarification represents a ‘definition’ role in Perez’s typology. But, as discussed below, we emphasize that the *process* of measurement, as much as the empirical findings, is conceptually productive.

33 These conceptual questions have important implications for how we think about the levels of relational equality currently realized across different countries. For example, in all countries in the European Social Survey 2012 the modal answer on the status ladder question was either 5 or 7. However, whereas in Portugal there are more people placing themselves below the mid-point than above it, in Sweden and Norway, almost no-one places themselves below 5. In the Netherlands, the vast majority place themselves at 7 or 8 (see Paskov and Richards 2021).

34 Perhaps not wholly contingent, since the relational exclusivist might consistently hold that certain distributive arrangements are constitutive of unequal regard. But such an account would need filling out, in a way that explains *why* the problem with these distributive forms is best cached out in relational terms. Otherwise, it is vulnerable to the charge of smuggling back in an independent commitment to distributive equality.

35 We thank an anonymous reviewer for this observation.

36 We do not find evidence of this type of inter-group difference in our data. But we did not set out to test this in our pre-analysis plan.

37 We use the terms ‘public opinion’ and ‘public attitudes’ broadly and interchangeably here, to refer to any normatively salient aspect of individual self-perceptions or other regarding views that might be revealed by survey methods. This usage contrasts with some empirical scholarship, where sense of individual identity or belonging is typically not regarded as an attitude.

38 For an example of a similar approach, but utilizing existing survey data to operationalize a philosophical ideal, see Baderin and Barnes (2020).

39 The approach we have sketched out above is much better developed in interdisciplinary work on deliberative democracy. See, for example, Gastil (2022).

40 See also Claassen (2011) on the ‘philosopher-investigator’ approach; and Swift (1999).
